# Inhibition of mutant IDH1 decreases D-2-HG levels without affecting tumorigenic properties of chondrosarcoma cell lines

**DOI:** 10.18632/oncotarget.3723

**Published:** 2015-03-30

**Authors:** Johnny Suijker, Jan Oosting, Annemarie Koornneef, Eduard A. Struys, Gajja S. Salomons, Frank G. Schaap, Cathelijn J.F. Waaijer, Pauline M. Wijers-Koster, Inge H. Briaire-de Bruijn, Lizette Haazen, Scott M. Riester, Amel Dudakovic, Erik Danen, Anne-Marie Cleton-Jansen, Andre J. van Wijnen, Judith V.M.G. Bovée

**Affiliations:** ^1^ Department of Pathology, Leiden University Medical Center, Leiden, The Netherlands; ^2^ Metabolic Unit, Department of Clinical Chemistry, VU University Medical Center, Neuroscience Campus Amsterdam, Amsterdam, The Netherlands; ^3^ Division of Toxicology, Leiden Academic Center for Drug Research, Leiden University, Leiden, The Netherlands; ^4^ Department of Orthopedic Surgery, Mayo Clinic, Rochester, NY, USA

**Keywords:** isocitrate dehydrogenase, d-2-hydroxyglutarate, chondrosarcoma, sarcoma, AGI-5198

## Abstract

Mutations in *isocitrate dehydrogenase 1 (IDH1)* and *IDH2* are found in a subset of benign and malignant cartilage tumors, gliomas and leukaemias. The mutant enzyme causes the production of D-2-hydroxyglutarate (D-2-HG), affecting CpG island and histone methylation. While mutations in *IDH1/2* are early events in benign cartilage tumors, we evaluated whether these mutations play a role in malignant chondrosarcomas. Compared to *IDH1/2* wildtype cell lines, chondrosarcoma cell lines harboring an endogenous *IDH1* (n=3) or *IDH2* mutation (n=2) showed up to a 100-fold increase in intracellular and extracellular D-2-HG levels. Specific inhibition of mutant IDH1 using AGI-5198 decreased levels of D-2-HG in a dose dependent manner. After 72 hours of treatment one out of three mutant *IDH1* cell lines showed a moderate decrease in viability, while D-2-HG levels decreased >90%. Likewise, prolonged treatment (up to 20 passages) did not affect proliferation and migration. Furthermore, global gene expression, CpG island methylation as well as histone H3K4, -9, and -27 trimethylation levels remained unchanged. Thus, while *IDH1/2* mutations cause enchondroma, malignant progression towards central chondrosarcoma renders chondrosarcoma growth independent of these mutations. Thus, monotherapy based on inhibition of mutant IDH1 appears insufficient for treatment of inoperable or metastasized chondrosarcoma patients.

## INTRODUCTION

Mutations in *isocitrate dehydrogenase 1* and *-2* (*IDH1* and -*2*) genes are found in acute myeloid leukemia (~20%) [1], gliomas (60-80%) [2, 3], cholangiocarcinomas (7-28%) [4-6] and in benign and malignant cartilage tumors [7-10]. Enchondroma is a benign cartilaginous tumor arising in the medulla of the bone. Enchondromas occur mostly solitary, while multiple enchondromas can be found in the non-hereditary Ollier disease or Maffucci syndrome [11, 12]. These syndromes are caused by somatic mosaic mutations in *IDH1* or *-2*. In 87% of these syndromic enchondromas and in 52% of sporadic enchondromas mutations are found, indicating that these are an early, driving and crucial event for the development of enchondroma [8, 9]. Malignant progression towards secondary central chondrosarcoma occurs in ~1% of solitary enchondromas and up to ~50% in patients with multiple enchondromas [13]. Central conventional chondrosarcomas carry mutations in *IDH1/2* in 38-70% of primary central chondrosarcomas (arising without a preexisting benign enchondroma) and in 86% of the secondary central chondrosarcomas [7-9].

Chondrosarcoma is the second most common primary malignant bone tumor and represents a heterogeneous group of tumors[14]. So-called dedifferentiation occurs in 10-15% of central chondrosarcomas [15]. Dedifferentiated chondrosarcoma is a highly malignant tumor characterized by a bimorphic histological appearance with distinct and abruptly separated areas of low grade chondrosarcoma juxtaposed to a high grade, non-cartilaginous sarcoma [16]. ~54% of the dedifferentiated chondrosarcomas contain mutations in *IDH1* or *IDH2* [8, 10].

The signaling pathways that regulate endochondral ossification are thought to also play a role in the development of enchondromas and chondrosarcomas [17]. Of these, Hedgehog signaling (Hh) is thought to be most important and constitutively active signaling is found in enchondromas and central chondrosarcomas [18, 19]. In gliomas, *IDH1/2* mutations are associated with active Hh signaling [20]. Isocitrate dehydrogenase is an enzyme involved in the conversion of isocitrate to α-ketoglutarate. Three isoforms of IDH are known. IDH1 is localized in the cytoplasm while IDH2 and IDH3 act in the mitochondria. Gain of function mutations are exclusively found on the arginine residues R132 in *IDH1* and R140 and R172 in *IDH2*. These mutations in *IDH-1* or -*2* lead to gain-of-function, by which the mutant enzyme acquires the activity to convert α-ketoglutarate into D-2-hydroxyglutarate (D-2-HG), but not to its enantiomer L-2-hydroxyglutarate (L-2-HG). The newly formed oncometabolite D-2-HG shows structural similarities with α-ketoglutarate, and as a result D-2-HG is able to competitively inhibit α-ketoglutarate dependent enzymes such as the ten-eleven translocation (TET) enzymes [21]. TET enzymes are involved in DNA demethylation [22-25]. Indeed, increased levels of D-2-HG have been found in cartilage tumors with an *IDH1* or *IDH2* mutation [8], and DNA hypermethylation was shown in enchondromas with an *IDH1/2* mutation [8, 9].

Genome-wide CpG methylation sequencing of chondrosarcoma biopsies revealed that *IDH1/2* mutations are associated with DNA hypermethylation at CpG islands but not at other genomic regions [26]. In addition, histone demethylases are also α-ketoglutarate dependent [21] and an increase in methylation of the histone H3 lysine residues was shown in knock-in mice with an *IDH1* R132H mutation [27]. Trimethylation of H3K4 positively regulates transcription, whereas trimethylation of H3K9 and H3K27 is associated with repression of transcription [28, 29]. Furthermore, mutations in *IDH1/2* are associated with stabilization of hypoxia inducible factor-1α (HIF1α) through an effect on the prolyl hydroxylases (PHD). Gliomas with an *IDH1/2* mutation show upregulation of HIF1α, whereas PHD activity is inhibited in artificial mutant *IDH1* cell lines [30].

The high prevalence of *IDH1/2* mutations in enchondroma and chondrosarcoma suggest a causal rather than a bystander role. This led us to investigate the function of the *IDH1/2* mutation in chondrosarcoma. Chondrosarcoma patients show a poor response to conventional chemotherapy and radiotherapy, and surgery is the mainstay of treatment. Alternative treatment strategies are urgently needed as no treatment options are currently available for patients with inoperable or metastatic disease. To evaluate the functional role of *IDH1/2* mutations we used a chondrosarcoma cell line panel including five *IDH1/2* wildtype, three endogenous *IDH1* mutant and two endogenous *IDH2* mutant cell lines, originating from conventional central as well as dedifferentiated chondrosarcomas. Remarkably glioma and leukemia cells harboring mutations in *IDH1* or *-2* cannot be maintained in culture, however we previously reported that chondrosarcoma cell lines retain these mutations [31] providing us with a model to functionally study the effects of *IDH1* or *-2* mutations in their naturally occurring context and to evaluate whether inhibition of mutant *IDH1/2* could be a potential treatment strategy.

## RESULTS

### Cell lines with an endogenous mutation in *IDH1* or -2 express the mutant allele

**Figure 1:**
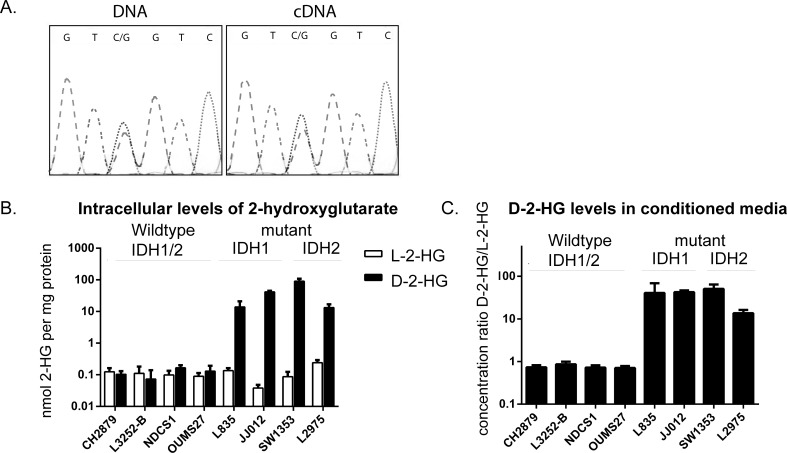
Comparison of the levels of D-2-HG and L-2-HG between *IDH1/2* wildtype and mutant *IDH1/2* chondrosarcoma cell lines **A**) *IDH1* mutation in cell line JJ012. Mutation status for all cell lines was confirmed and the expression of the mutant allele was shown by Sanger sequencing of the cDNA. **B**) intracellular levels of D-2-HG and L-2-HG normalized to total protein concentration. Elevated levels of D-2-HG up to a 1000 fold were observed in mutant *IDH1/2* cell lines compared to the *IDH1/2* wildtype chondrosarcoma cell lines. **C**) Elevated levels of D-2-HG were also found in the conditioned media, D-2-HG levels were normalized to L-2-HG concentration. Error bars represent mean ± SD of measurements in three different passages.

Sequencing for mutations in exon 4 of *IDH1* and -*2* in the cell lines confirmed the previously reported heterozygous mutations in JJ012 (*IDH1* R132G), L835 (*IDH1* R132C), SW1353 (*IDH2* R172S), HT1080 (*IDH1* R132C) and the homozygous R172W *IDH2* mutation in L2975. Moreover, in all cell lines the mutant allele was expressed, as shown by sequencing of the corresponding cDNA samples (example shown in Figure 1A).

### Mutant *IDH1* or -2 cell lines have increased levels of D-2-HG intra- and extracellularly

Intracellular levels of D-2-HG as determined by LC-MS/MS were elevated up to a 1000-fold in cell lines with *IDH1* or *-2* mutations as compared to cell lines wildtype for *IDH1/2* (Figure 1B). Measurements of extracellular D/L-2-HG levels in the culture media revealed that the ratio between D-2-HG and L-2-HG was elevated up to a 100-fold in mutant *IDH1/2* cell lines (Figure 1C). No elevated levels of the enantiomer L-2-HG were found in mutant *IDH1* or -*2* cell lines. Levels of D-2-HG did not differ between cell lines with a mutation in *IDH1* versus those with a mutation in *IDH2*.

### Cell lines with mutations in *IDH1* or -2 have a different methylome as compared to wildtype cell lines

**Figure 2:**
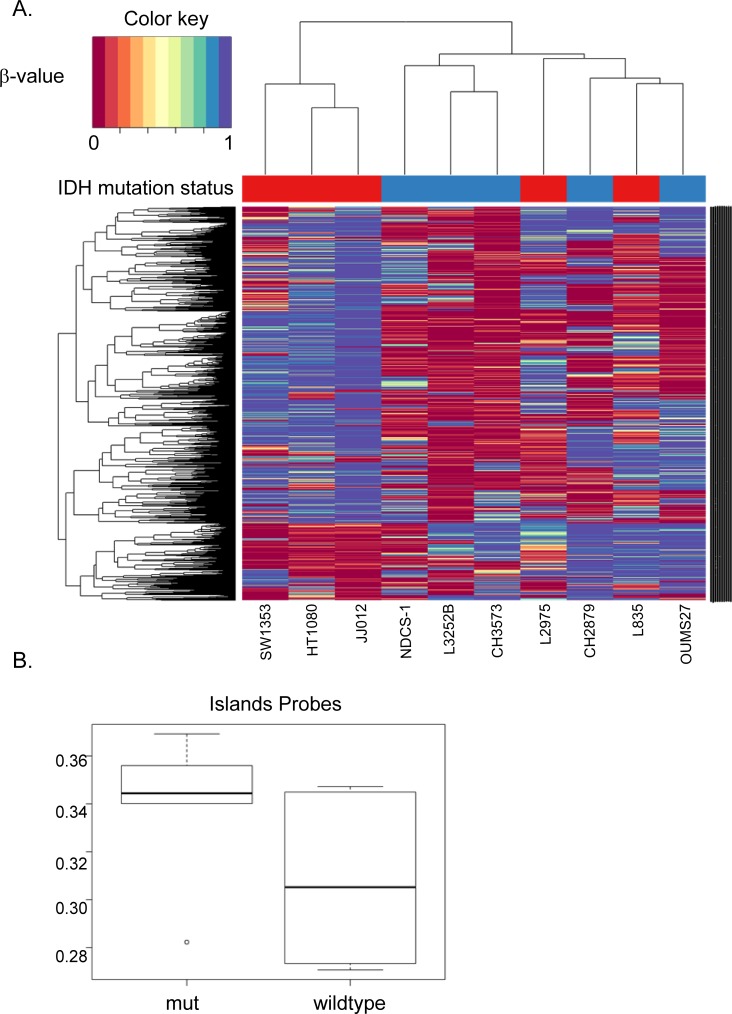
Unsupervised hierarchical clustering of the top 2000 most differentially methylated CpG sites in CpG islands (**A**) Unsupervised hierarchical clustering for the top 2000 most differentially methylated CpG sites resulted in a wildtype cluster (blue samples), a mutation specific cluster (red samples) and a cluster with a mixture of wildtype and mutant *IDH1/2* cell lines. (**B**) CpG islands are known to be located in promoter regions. As expected, methylation in the CpG islands was higher in the mutant *IDH1/2* cell lines compared to the wildtype counterparts.

Since mutations in *IDH1* were shown to cause global hypermethylation in tumors [9, 26, 32], we assessed the methylome in the chondrosarcoma cell lines. Unsupervised hierarchical clustering for the top 2000 most differentially methylated CpG sites resulted in a cluster containing *IDH1/2* wildtype and a cluster containing mutant *IDH1/2* cell lines (Supplementary Figure 1A). Unexpectedly, when all 480 000 probes are included, comparison of the mean β-values between mutant *IDH1/2* and *IDH1/2* wildtype cell lines suggested global hypermethylation of the *IDH1/2* wildtype cell lines (Supplementary Figure 1B). When analyzing the different probes separately, only CpG islands probes showed hypermethylation in the chondrosarcoma cell lines with an *IDH1/2* mutation (Figure 2B), whereas the shores (regions flanking the CpG islands) and shelves (regions flanking the shores) show hypermethylation in the *IDH1/2* wildtype cell lines (Supplementary Figure 1B). Since CpG islands are known to be located in promoter regions and because *IDH1/2* mutations are associated with DNA hypermethylation at CpG islands but not at other genomic regions [26], we focused on the probes that were CpG island specific. Unsupervised hierarchical clustering of CpG islands specific probes resulted in three clusters. The first containing mutant *IDH1/2* cell lines only, the second containing *IDH1/2* wildtype cell lines and the third cluster contained a mixture of wildtype and mutant *IDH1/2* cell lines (Figure 2A).

### Inhibition of mutant *IDH1* does not affect viability or migration of mutant *IDH1* cell lines

**Figure 3:**
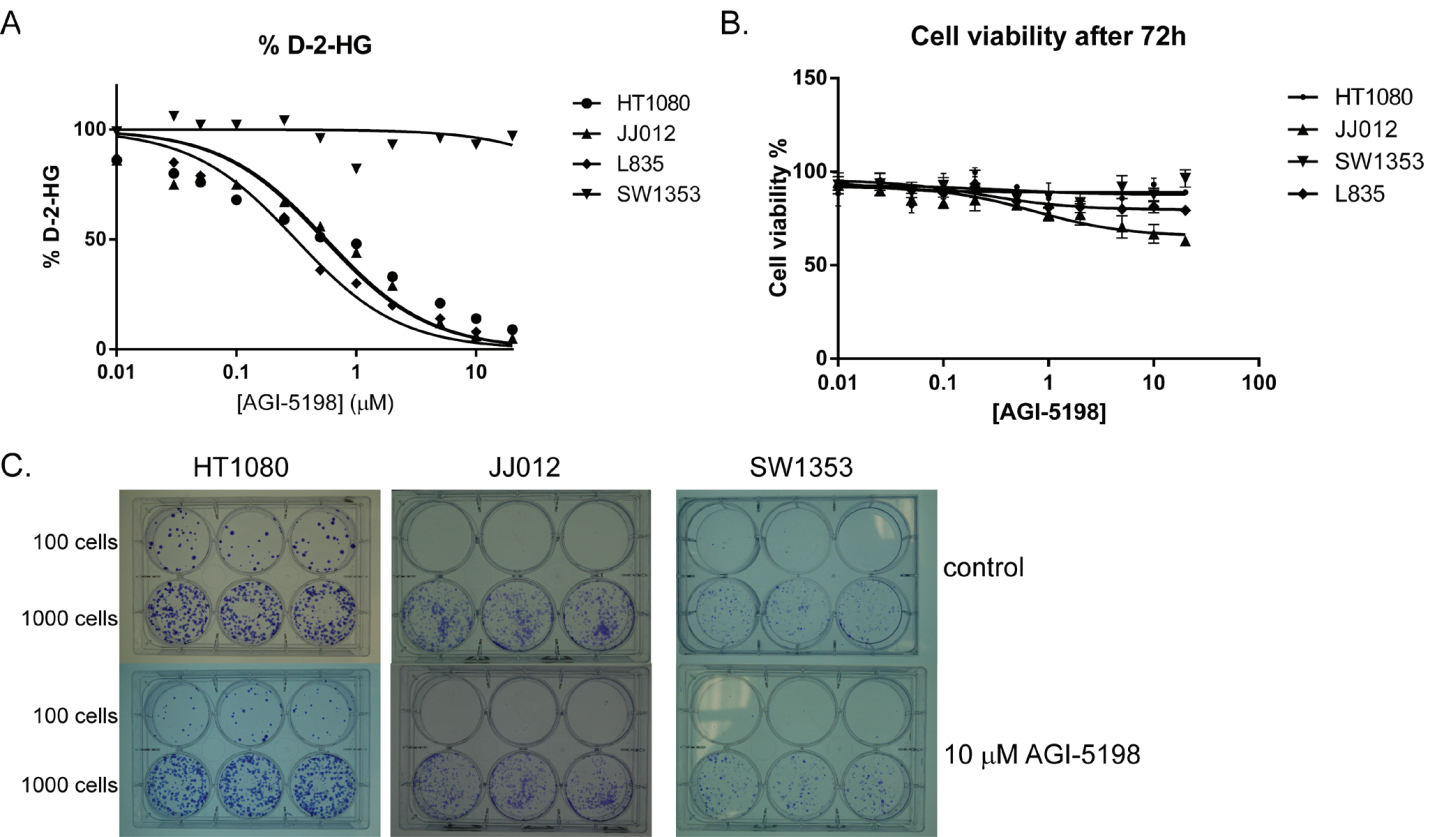
Inhibition of mutant IDH1 for 72 hours using AGI-5198 in chondrosarcoma cell lines (**A**) Specific inhibition of mutant IDH1 decreased D-2-HG levels in the culture medium up to 90% in the mutant *IDH1* cell lines L835, HT1080 and JJ012, whereas treatment with AGI-5198 did not affect D-2-HG levels in the mutant *IDH2* cell line SW1353. (**B**) Interestingly, inhibition of D-2-HG levels up to 90% had a minor effect on viability (approximately 65% and 80% viability left in respectively JJ012 and L835 after 72 hours). Error bars represent mean ± SD of measurements in triplicates. (**C**) A colony forming assay was performed for the *IDH1* mutant cell lines HT1080 and JJ012 and the *IDH2* mutant cell line SW1353. Cells were seeded in a density of 100 cells and 1000 cells in triplicate. Treatment with 10 μM of AGI-5198 did not affect colony formation after 10 days.

**Figure 4:**
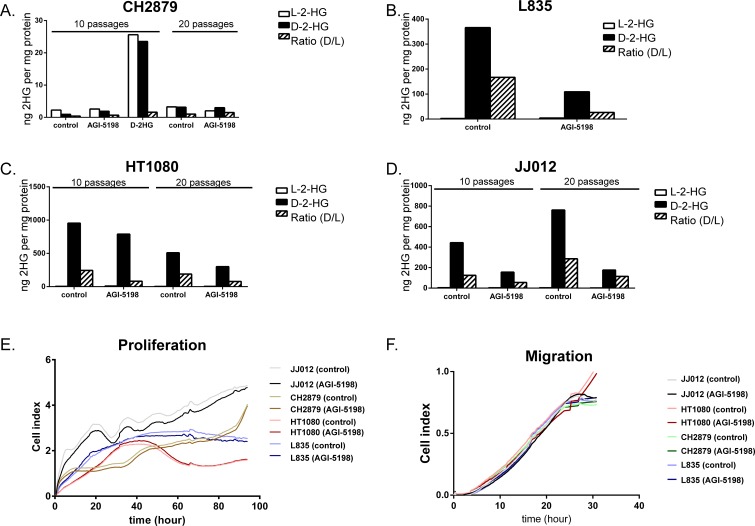
Prolonged treatment of *IDH1/2* wildtype and *IDH1* mutant cell lines with mutant IDH1 inhibitor AGI-5198 and metabolite D-2-HG Prolonged treatment with 1.5 μM IDH1 mutant inhibitor AGI-5198 did not affect intracellular D-2-HG levels of the *IDH1/2* wildtype cell line CH2879 after 10 and 20 passages, while treatment with D2HG resulted in increased levels of L2HG and D2HG intracellularly (**A**). Chondrosarcoma cells harboring an *IDH1* mutation (L835 (**B**), HT1080 (**C**), JJ012 (**D**)) show decreased levels of intracellular D-2-HG compared to untreated cells after continuous treatment with 1.5 μM AGI-5198 for 10 and 20 passages, respectively. For L835, due to slower growth rate, only 10 passages were evaluated. The xCelligence assay demonstrated no effect of prolonged inhibition of mutant IDH1 on proliferation (**E**) or migration (**F**). Graphs show one representative experiment out of three experiments.

To assess the role of mutant IDH1 in chondrosarcoma cell proliferation, we incubated the cell lines with the IDH1 mutant inhibitor AGI-5198 for 72 hours. Indeed the levels of D-2-HG in culture media decreased in a dose-dependent manner in the mutant *IDH1* cell lines JJ012 (R132G), HT1080 (R132C) and L835 (R132C) with IC50s of 0.7 μM, 0.5 μM and 0.35 μM, respectively (Figure 3A). As expected, D-2-HG levels of the mutant IDH2 SW1353 cell line were not affected (Figure 3A). Interestingly, even at doses that led to >90% decrease in D-2-HG (20 μM AGI-5198 for 72 hours), only a minor effect on cell viability using a WST-1 assay was seen (approximately 65% viability left in JJ012 after 72 hours)(Figure 3B). Furthermore, mutant IDH1 inhibition did not influence the colony forming capacity of the mutant *IDH1* cell lines HT1080 and JJ012 and the mutant *IDH2* cell line SW1353 after 10 days of treatment, whereas L835 was not able to form colonies in both the control and the AGI-5198 conditions (Figure 3C). Since glioma cells transfected with mutant *IDH1* need 20 passages to show an effect on methylation [33], we treated the mutant *IDH1* cell lines JJ012, HT1080 and the *IDH1/2* wildtype cell line CH2879 for up to 20 passages. Due to its slower growth rate, L835 was treated for up to 10 passages. As expected, treatment of CH2879 with 5mM D-2-HG for 10 passages showed a slight increase of intracellular levels of D-2-HG (10 times compared to control) (Figure 4A). We confirmed that after treatment with 1.5 μM AGI-5198 for 10 and 20 passages intracellular levels of D-2-HG were decreased in mutant *IDH1* cell lines L835, HT1080 and JJ012 compared to untreated cells (Figure 4B-4D). However, the xCelligence assay did not show any effect of AGI-5198 treatment on proliferation or migration (Figure 4E-4F). Moreover, the ability of *IDH1* mutant cells to migrate in 3D extracellular matrix scaffolds was not affected by treatment with 1.5 μM AGI-5198 (Supplementary Figure 2). Indeed, migration was not dependent on the *IDH1/2* mutation status, since mutant *IDH1* and mutant *IDH2* cell lines as well as the *IDH1/2* wildtype cell line showed migration in the timeframe of this experiment.

### Long term inhibition of mutant *IDH1* does not significantly affect CpG island methylation, histone trimethylation of H3K4, H3K9 and H3K27 or gene expression

**Figure 5:**
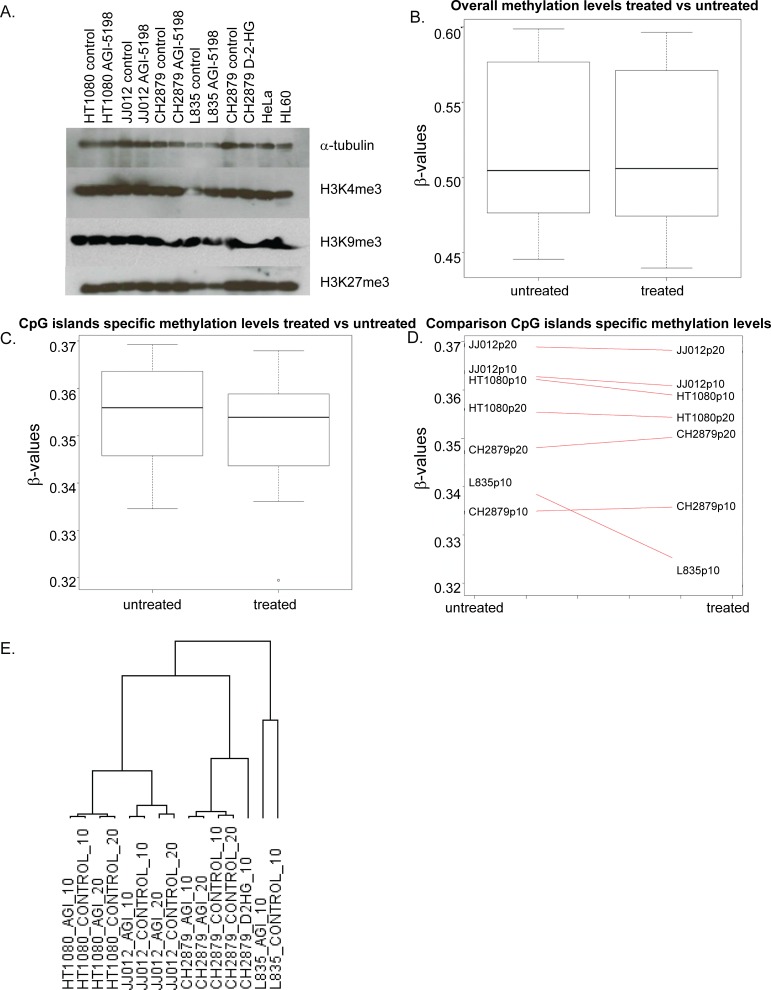
The effect of long term mutant IDH1 inhibition on histone and DNA methylation and gene expression (**A**) Western blot showing levels of trimethylation of Histone H3 K4, K9 and K27 after treatment with 1.5 μM AGI5198 or 5 mM D-2-HG for 10 or 20 passages. Treatments did not have an effect on trimethylation of each of the lysines in the mutant *IDH1* chondrosarcoma cell lines. (**B**) Global methylation levels after prolonged treatment with AGI-5198 remain unchanged, with overall mean β-values of both treated and untreated samples of approximately 0.5. (**C**) When analyzing the CpG islands specific probes separately, minimal demethylation of the genome is seen. (**D**) Differences in CpG island specific methylation levels are predominantly seen in the L835 cell line treated for 10 passages. (**E**) Unsupervised hierarchical clustering of all expressed genes in the RNAseq dataset showed that the expression profiles of the cell lines are not dramatically changed by the treatment, since expression profiles of the treated cell lines are more similar to their untreated counterparts than to other treated cell lines. Samples were labelled according to the cell line, followed by the treatment (AGI=treated with 1.5 μM AGI-5198; CONTROL=treated with DMSO) and the number of passages (10=10 passages of continuous treatment; 20=20 passages of continuous treatment).

Since mutations in *IDH1* are associated with inhibition of demethylases [21, 27], we tested the effect of prolonged treatment with AGI-5198 and D-2-HG on trimethylation levels of H3K4, H3K9 and H3K27. The mutant *IDH1* chondrosarcoma cell line L835, treated with 1.5 μM AGI-5198 for 10 passages, and the mutant *IDH1* cell lines HT1080 and JJ012, treated for 20 passages, did not show any difference in lysine trimethylation of these specific histone marks (Figure 5A).

Comparison of the mean overall methylation levels did not show any difference in global methylation levels in treated versus untreated cell lines (Figure 5B). When focusing on the CpG islands specific probes, a slight decrease in overall CpG island specific methylation was seen (Figure 5C). When assessing the treated and untreated pairs separately, demethylation of CpG island specific probes was predominantly seen in L835 treated for 10 passages (Figure 5D).

**Figure 6:**
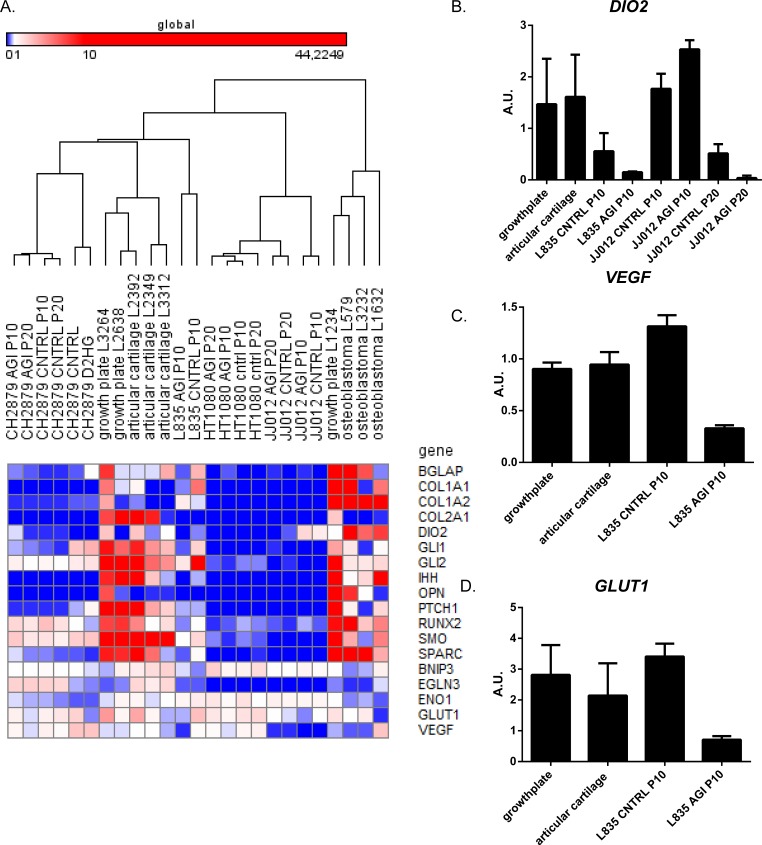
The effect of long term mutant IDH1 inhibition on hedgehog signaling, HIF1α signaling and chondrogenic and osteogenic differentiation markers. ( **A**) qPCR results shown in a heatmap of expression levels of genes involved in Hedgehog signaling, HIF1α signaling and osteogenic or chondrogenic differentiation. Comparison of the expression levels of genes involved in Hedgehog signaling, as well as osteogenic and chondrogenic differentiation markers revealed lower expression levels in the cell lines compared to 3 growth plates, 3 articular cartilage and 3 osteoblastomas. HIF1α target genes showed comparable expression levels between cell lines and controls. No differences in expression levels were observed after treatment with D-2-HG. The color scheme was set on global with the lowest expression levels in blue (RKPM value starts at 0), with the intermediate scale set on white (RKPM value starts at 1) and the highest expression levels in red (RKPM value starts at 10). (**B**) Expression levels of *DIO2* in samples after mutant IDH1 inhibition and in the growth plate and articular cartilage controls. DIO2 expression levels decreased upon inhibition of mutant IDH1 in L835 after 10 passages and in JJ012 after 20 passages. (**C**) Expression levels of *VEGF* were decreased after mutant IDH1 inhibition in L835. (**D**) Expression levels of *GLUT1* were decreased after mutant IDH1 inhibition in L835.

Studying global gene expression using RNAseq revealed that prolonged inhibition of mutant IDH1 did not dramatically change global gene expression. Unsupervised hierarchical clustering showed that the expression profiles of the treated cell lines are more similar to their untreated counterparts, than to other treated cell lines (Figure 5E). We identified 2 genes that were differentially expressed (FDR<0.05) after 10 passages of continuous treatment (Table 1), whereas after 20 passages, 10 genes were differentially expressed (FDR<0.05) (Table 2), without any overlap between 10 and 20 passages of continuous treatment. Interestingly, gene expression in HT1080 is more stable than in L835 or JJ012, since after 10 or 20 passages of continuous treatment, genes with a FDR<0.05 are shown to be influenced exclusively in L835 and JJ012 (Tables 1 and 2). We chose *COL1A2* and *DIO2,* based on their role in differentiation and chondrocyte biology, for further validation using qPCR [34]. Indeed, expression of *DIO2* decreased upon inhibition of mutant IDH1 in L835 after 10 passages and in JJ012 after 20 passages of continuous mutant IDH1 inhibition (Figure 6B). Expression of COL1A2 was very low in all cell lines as compared to osteoblastoma and normal growth plate controls (data not shown).

**Table 1:** Differentially expressed genes after 10 passages in cell lines with and without inhibition of mutant IDH1 using AGI-5198 (FDR<0.05) The expression levels are shown in Reads per Kilobase per Million (RPKM)

Chr	GeneID	HT1080 p10 AGI-5198 (RKPM)	HT1080 p10 control (RKPM)	JJ012 p10 AGI-5198 (RKPM)	JJ012 p10 control (RKPM)	L835 p10 AGI-5198 (RKPM)	L835 p10 control (RKPM)
Chr7	*CHRM2*	0	0	0	0	2.391	0.011
Chr6	*SIM1*	0	0	0.024	0	0.848	0.063

**Table 2:** Differentially expressed genes after 20 passages of continuous treatment with AGI-5198 (FDR<0.05) The expression levels are shown in Reads per Kilobase per Million (RPKM)

Chr	GeneID	HT1080 p20 AGI-5198 (RKPM)	HT1080 p20 control (RKPM)	JJ012 p20 AGI-5198 (RKPM)	JJ012 p20 control (RKPM)
Chr7	*COL1A2*	0.136	0.052	75.973	38.604
Chr19	*RFPL4A*	0.065	0.249	1.203	5.090
Chr9	*PTGDS*	0	0	33.285	11.424
Chr13	*POSTN*	0.070	0	0.785	0.190
Chr5	*ADAMTS12*	0.053	0.097	2.230	5.086
Chr14	*IFI27*	1.398	1.042	108.563	49.819
Chr9	*CLIC3*	0.832	0.418	37.160	17.884
Chr1	*RYR2*	0.226	0.372	0.648	1.296
Chr14	*DIO2*	0.074	0.080	3.360	10.739
Chr6	*ROS1*	0.028	0.081	0.169	0.457

Analysis of gene expression using qPCR revealed that osteogenic and chondrogenic differentiation related genes (*COL1A1, COL2A1, RUNX2, BGLAP, OPN, SPARC*) were only very lowly expressed in the cell lines before and after treatment, in comparison to the normal cartilage, growth plate and osteoblastoma controls (Figure 6A). The target genes of HIF1α (*BNIP3, EGLN3, ENO1, GLUT1, VEGF)* showed comparable expression levels between cell lines and controls. Treatment with D-2-HG did not influence the gene expression of these HIF1α target genes (data not shown), whereas treatment with AGI-5198 led to a decrease in the expression of *VEGF* and *GLUT1* in L835 only (Figure 6C and 6D). Expression levels of genes involved in hedgehog signalling (*IHH, SMO, PTCH1, GLI1, GLI2*) were variable and no consistent change in expression was seen upon inhibition of mutant IDH1 (data not shown).

## DISCUSSION

The high prevalence of *IDH1* or *-2* mutations in enchondroma and chondrosarcoma, suggesting a causal rather than a bystander role in tumorigenesis, led us to investigate the function of these mutations in chondrosarcoma. Since glioma and leukemia cells harboring mutations in *IDH1* or *-2* cannot be maintained in culture, previous studies in these tumor types used artificial models to study the role of *IDH1/2* mutations. We here use a unique panel of chondrosarcoma cell lines harboring endogenous *IDH1/2* mutations that were retained during culturing [9, 31]. We confirmed that all cell lines with an endogenous mutation in *IDH1 or -2* expressed the mutant allele, which was associated with elevated intracellular and extracellular levels of D-2-HG. Our chondrosarcoma cell line panel contained one homozygous mutant *IDH2* cell line (L2975) that, contradictory to what has been shown in the anaplastic astrocytoma WHO grade III cell line IMA [35], revealed elevated levels of D-2-HG comparable to heterozygous mutant *IDH1/2* cell lines. Thus, our panel provides us with an excellent model to functionally study the consequences of *IDH1 or -2* mutations and to evaluate whether inhibition of mutant *IDH1* could be a potential treatment strategy.

To inhibit the mutant IDH1 protein we used the commercially available small molecule inhibitor AGI-5198, which is a potent and selective inhibitor of IDH1 with mutations on position R132 [36]. Inhibition of the mutant IDH1 protein indeed decreased the levels of D-2-HG in a dose-dependent manner. In addition to the previously reported *IDH1* R132H and R132C mutations [37], we here show that D-2-HG levels of *IDH1* R132G mutant cells can also be decreased using AGI-5198. The selectivity of AGI-5198 was confirmed in the mutant *IDH2* cell line SW1353, where treatment did not affect D-2-HG levels. Although inhibition of mutant IDH1 decreased levels of D-2-HG up to 90%, only one out of three *IDH1* mutant chondrosarcoma cell lines (JJ012) showed a minor effect on viability. In addition, prolonged treatment during 10 or 20 passages also did not affect proliferation or migration in monolayer cultures nor in a 3D collagen scaffold model. Moreover, the colony forming capacity of the mutant *IDH1* cell lines after 10 days was not affected by treatment with 10 μM AGI-5198. These results are in contrast to results in glioma since Rohle et. al. showed that AGI-5198 impaired soft-agar colony formation in the transformed glioma cell line TS603. Moreover, an effect on colony formation after knockdown of *IDH1* with shRNAs was shown [37]. A possible explanation for this difference might be that the shRNA targets both the wildtype and the mutant allele, which are expressed both as we show in Figure 1A. In addition, the difference may be explained by the difference in tumor types, or by a difference in model systems used (endogenous mutation versus artificially induced mutation). Our results suggest that mutations in *IDH1/2*, while being essential as an early event in the development of benign enchondroma, are not essential anymore after progression towards high grade chondrosarcoma. It is likely that other, additional genetic events occurred during tumor progression that have taken over driving chondrosarcoma cell proliferation.

Since the elevated levels of the oncometabolite D-2-HG caused by the mutation are reported to affect CpG island hypermethylation and histone methylation thereby affecting gene expression, as well as to interfere with HIF1α signaling, we next investigated the effect of inhibition of mutant IDH1 on these cellular processes in our cell lines. Mutant IDH1/2 causes hypermethylation of the genome via inhibition of α-ketoglutarate dependent enzymes in AML [27], gliomas [38], enchondromas [9] and chondrosarcomas [26]. Most studies focused on CpG island regions. In this study, we used the Illumina HumanMethylation 450k Beadchip array covering CpG islands, shores and shelves. Even though it is well known that prolonged culturing affects global methylation levels in cell lines [39, 40], we were able to show a different methylome in *IDH1/2* mutant and *IDH1/2* wildtype chondrosarcoma cell lines. Interestingly, only CpG islands probes show hypermethylation in the chondrosarcoma cell lines with an *IDH1/2* mutation, whereas the shores and shelves show hypermethylation in the *IDH1/2* wildtype cell lines. When all 480 000 probes were included, comparison of the mean β-values between mutant *IDH1/2* and *IDH1/2* wildtype cell lines showed general hypermethylation of the *IDH1/2* wildtype cell lines. Thus, even though cell lines have been in culture for a long time, the mutation in *IDH1/2* is associated with increased levels of methylation at the CpG islands, and decreased levels of methylation at other areas in the genome.

In TS603 glioma xenografts treated with AGI-5198 for two weeks Rohle et. al. showed no statistically significant change in the genome wide distribution of DNA methylation [37]. Likewise, we observed no significant change in gene expression nor in genome wide methylation distribution after continuous inhibition of mutant IDH1 during 10 or 20 passages. However, when focusing only on the CpG islands, methylation slightly decreased, an effect which was predominantly caused by alterations in L835. L835 is a slowly growing chondrosarcoma cell line that we previously generated in our lab [31], which has had a much lower number of passages in culture as compared to the widely used cell lines JJ012 and HT1080. Thus, L835 might resemble the primary tumor more than the cell lines that were cultured for a longer period of time. Indeed, it is therefore of interest that L835 is the only cell line demonstrating a decrease in CpG island specific methylation as well as a decrease in expression of HIF1α downstream targets *GLUT1* and *VEGF*, upon inhibition of mutant IDH1. On the other hand, HT1080, formally derived from a “fibrosarcoma of bone” but in retrospect presumably representing dedifferentiated chondrosarcoma based on its *IDH1* mutation, showed no alterations in gene expression, nor in CpG island methylation levels. Not surprisingly, this cell line does not express any of the differentiation markers either. It is tempting to speculate that HT1080 obtained many additional molecular alterations upon dedifferentiation (i.e. fibrosarcomatous change in conventional chondrosarcoma) and prolongation in culture, thereby rendering the *IDH1* mutation non-essential.

In addition to downregulation of HIF1α downstream targets in L835 upon inhibition of mutant IDH1, RNAseq analysis revealed downregulation of the *deiodinase iodothyronine type 2 (D2) gene DIO2*, which was confirmed using qPCR. The gene product of *DIO2* catalyzes the conversion of intracellular inactive thyroid hormone (T4) to active hormone (T3) [34]. T3 is involved in growth plate chondrocyte maturation [41, 42]. This suggests that a mutation in *IDH1/2* would upregulate DIO2, which would increase the levels of T3 thereby promoting chondrocyte maturation.

In summary, both short term as well as long term continuous inhibition of endogenous mutant IDH1 in three different chondrosarcoma cell lines show that although D-2-HG levels are decreased by the treatment, there is no significant effect on proliferation, migration (both in monolayer as well as in 3D), CpG island methylation or histone trimethylation of H3K4, H3K9 and H3K27. At the gene expression level we show inhibition of HIF1α target genes and of *DIO2* in L835. The latter finding is novel and suggests a role of DIO2 and thyroid signaling in the development of enchondroma and chondrosarcoma, which would warrant further study. Our results suggest that while mutations in *IDH1 or-2* are an early event in tumorigenesis, chondrosarcoma is not dependent on mutant *IDH1* anymore. Thus, other events are likely to be involved in chondrosarcoma progression, in which the mutation in *IDH1 or -2* no longer seems to be a driver mutation. Even though promising results are being reported for IDH1 inhibitors in early clinical trials in leukemia [43, 44], our results indicate that monotherapy using mutant IDH1 inhibition may not be sufficient to use as a treatment option for patients with inoperable or metastatic central chondrosarcomas harboring this mutation.

## MATERIALS AND METHODS

### Compounds

The specific IDH1 mutant inhibitor AGI-5198 (IDH-C35; Xcess Biosciences, Inc.) was dissolved in DMSO. D-2-hydroxyglutarate (RC402; PepTech Corporation) and L-2-hydroxyglutarate (90790; Sigma-Aldrich) were dissolved in PBS.

### Cell lines and culturing

The chondrosarcoma cell lines CH2879 [45], NDCS-1 [46], OUMS27 [47], JJ012 [48], SW1353 (ATCC), L3252, L2975, and L835 [31] were used. In addition, we used HT1080 (ATCC) as it was originally reported as a fibrosarcoma of bone, and is now known to harbor a mutation in *IDH1*. Since fibrosarcoma is a diagnosis of exclusion [16], in retrospect this tumor probably reflects a dedifferentiated chondrosarcoma. Cells were cultured in RPMI1640 (52400-025; Gibco, Invitrogen Life-Technologies, Scotland, UK) supplemented with 1% penicillin/streptomycin (P/S) (100 U/ml), and 10% heat-inactivated fetal bovine serum (F7524; Sigma-Aldrich). Cells were grown at 37°C in a humidified incubator with 95% air and 5% CO2. Cell lines were tested for mycoplasma once a month via PCR [49]. STR typing was performed on all cell lines using the PowerPlex 1.2 system (Promega Benelux BV, Leiden, The Netherlands) to confirm their identity.

### Analysis of *IDH1* and -2 mutation status

Both genomic DNA and cDNA were used for mutation analysis. DNA isolation was performed using the wizard genomic DNA purification kit (Promega, Madison, WI) according to the manufacturer's instructions. RNA was isolated using TRIzol (Invitrogen, cat nr 15596-018), and purified with the RNeasy mini kit (Qiagen, cat nr 74104). cDNA synthesis was performed with AMV-RT enzyme (Roche Diagnostics, Cat. nr. 109118), oligodTs and random hexamer primers were used. *IDH1* and *-2* mutation status was confirmed on DNA, as described previously [9]. PCR reactions were carried out in a total volume of 25 μl and contained either 100 ng DNA or 5 μl of 25x diluted cDNA (equivalent to 40 ng total RNA). After each PCR run, melting curves were inspected in order to check the formation of a single product. The PCR products were purified using the QIAquick PCR Purification Kit (Qiagen, Hilden, Germany) according to the manufacturer's manual and eluted in a final volume of 40 μl. Bidirectional Sanger sequencing was performed by Macrogen (Amstelveen, The Netherlands) using M13 general primers. The sequence results were evaluated using Mutation Surveyor software (Soft-Genetics).

### D/L-2-hydroxyglutarate measurements

Extracellular D/L-2-hydroxyglutarate was measured using an aliquot (1 mL) of culture medium conditioned for 72 hours. Intracellular D/L-2-hydroxyglutarate was measured in cell lysates. The cells were washed with PBS and taken up in Hanks balanced salt solution (HBSS). The cells were lysed by three cycles of freeze-thawing in dry ice. To distinguish between D-2-HG and L-2-HG, samples were measured via LC-MS/MS as described elsewhere [50]. Intracellular D/L-2-HG measurements were normalized to the total protein concentration. For the extracellular D/L-2-HG measurements, the levels of D-2-HG were normalized to the amount of L-2-HG since the L-2-HG levels were constant for all cell lines. Protein concentrations were measured using the Biorad Dc Assay (Bio-Rad Laboratories B.V., Veenendaal, The Netherlands) as described in the manufacturer's manual.

### Western blotting

Whole cell lysates were made using the HOT-SDS protocol, lysis buffer contained complete inhibitors and phosphoStop. Proteins were separated by SDS-PAGE and transferred to a polyvinylidene difluoride membrane by a wet blotting system according to manufacturer's manual (Bio-Rad Laboratories B.V., Veenendaal, The Netherlands). After blocking with 5% non-fat dry-milk, the primary antibody, H3K4me3 (Millipore, 07-473); H3K9me3 (Abcam, ab8898); H3K27me3 (Millipore, 07-449) was incubated overnight at 4°C, at dilutions of 1:1000, 1:2000 and 1:2000 respectively. Afterwards the membrane was washed with PBS/0,05% Tween-20, and the appropriate secondary antibody was incubated for 30 minutes at room temperature. After a final washing step, the membrane was developed using the ECL system (Thermo Scientific, #32209) according to the manufacturer's protocol.

### Viability assay

Cells were seeded in 96 well plates and allowed to adhere overnight. For SW1353 and JJ012 5000 cells per well were seeded, for HT1080 and L835 10 000 cells and 15 000 cells, respectively. After 72 hours incubation with AGI-5198, a WST-1 assay (REF: 11 644 807 001, Roche) was performed according to the manufacturer's instruction and analyzed with the Victor3V, 1420 Multilabel plate reader (Perking Elmer, NL). Assays were performed in triplicate for three independent experiments. For long term treatment with AGI-5198 and D-2-HG, cells were treated for 10 or 20 passages with 1.5 μM AGI-5198. Medium with compounds or solvent controls were refreshed twice a week.

### Real time proliferation and migration assays

The RTCA xCelligence system (Acea, http://www.aceabio.com) was used to study either proliferation or migration in real time. Proliferation was monitored for 72 hours in E-plates. For the migration assay, SIM plates were used with the lower compartment filled with RPMI1640 with 20% FCS as a chemoattractant, cells were plated in the upper compartment in RPMI1640 only.

### 3D migration assay

Cells were pretreated for 72 hours with either mutant IDH1 inhibitor 10 μM AGI-5198 or DMSO, after which cells were trypsinized and collected. The migration assay was performed as described elsewhere [51]. Pictures were taken one hour, one day, two days and three days after injection of cells into the matrix. Pictures were taken using the Motic Moticam 3 CMOS 3.0MP Color Digital Camera and the motic images plus 2.0 ML Software.

### Colony forming assay

HT1080, JJ012, L835 and SW1353 cells were trypsinized and 100 and 1000 cells were seeded in a 6-well format. Cells were allowed to form colonies over 10 days and were either treated with DMSO or 10 μM AGI-5198 on day 0. Afterwards, cells were fixed and stained with 6.0% glutaraldehyde and 0.5% crystal violet.

### Quantitative real time PCR

PCR reactions were carried out and relative gene expression levels were normalized for DNA input using the housekeeping genes *PPIA*, *CPSF6* and *GPR108,* as described previously [52]. We used Real Time-PCR to evaluate the effect of D-2-HG and mutant IDH1 inhibition on Indian hedgehog signaling (*IHH, SMO, PTCH1, GLI1, GLI2*), HIF1α signaling (*GLUT1*, *BNIP3*, *EGLN3*, *ENO1*, *VEGF*), and differentiation (*COL1a1*, *COL2a1*, *RUNX2*, *BGLAP*, *OPN*, *SPARC*). In addition, we evaluated the expression of *COL1A2* and *DIO2*. PCR-primers are shown in Supplementary Table 1. Bio-Rad CFX Manager software was used to analyze data from the PCR experiments. As normal controls for Hedgehog signalling and cartilaginous differentiation we included RNA isolated from normal articular cartilage (n=3) and normal growth plate cartilage (n=3). For genes involved in osteogenic differentiation we included RNA derived from osteoblastoma (n=3). To give an overview of the expression levels of each of the genes and samples, a heatmap was made using the Gene-E tool (http://www.broadinstitute.org/cancer/software/GENE-E/index.html).

### Methylation analysis

Genomic DNA was bisulfite-converted using the EZ DNA Methylation Gold Kit (Zymo Research) and used for microarray-based DNA methylation analysis, performed at ServiceXS (ServiceXS B.V., Leiden, The Netherlands) using the HumanMethylation450 BeadChip array (Illumina, Inc., San Diego, CA, U.S.A). This array interrogates over 450,000 CpG sites representing about 99% of the RefSeq genes. The bisulfite-converted DNA was processed and hybridized to the HumanMethylation450 BeadChip (Illumina, Inc.), according to the manufacturer's instructions. Analysis was performed using ‘R project’. Samples were normalized using BMIQ [53].

### Next generation RNA sequencing analysis

RNA was isolated using TRIzol (Invitrogen, cat nr 15596-018). High througput sequencing of mRNAs was performed as previously described in detail [54]. This procedure uses libraries obtained using the TruSeq RNA Sample Prep Kit v2 (Illumina) and indexes from the standard TruSeq Kits (12-Set A and 12-Set B) that were incorporated at the adaptor ligation step for multiplex sample loading on the flow cells. Purified and adapter-modified DNA fragments were enriched by 12 cycles of PCR using primers included in the Illumina Sample Prep Kit. The Agilent Bioanalyzer DNA 1000 chip was used for quality control to determine DNA concentrations and size distributions, followed by validation of the quantification using Qubit fluorometry (Invitrogen). Samples were examined as paired-end reads on an Illumina HiSeq 2000 platform using TruSeq SBS sequencing kit version 3, HCS v2.0.12 data collection software and Illumina's RTA version 1.17.21.3 for base-calling. RNA-Seq data were analyzed using a the MAPRSeq v.1.2.1 system and a data pipeline that includes alignment with TopHat 2.0.6 HTSeq software and EdgeR 2.6.2 [55-57]. Analyses of the RNA in R using the EdgeR package were performed using the negative binomial distribution. The dispersion factor was estimated in EdgeR using a weighted combination of both a gene-specific dispersion effect and a common dispersion effect calculated from all genes. To determine differentially expressed genes between the treated and untreated samples the threshold for false discovery rate (FDR) was set on 0.05. Hierarchical clustering of samples was performed using the Gene-E tool.

## SUPPLEMENTARY MATERIAL FIGURES AND TABLE


