# Supplementary material for: Inhibition of mutant IDH1 decreases D-2-HG levels without affecting tumorigenic properties of chondrosarcoma cell lines
Source: Oncotarget. 2015 Mar 30;6(14):12505–19. doi: 10.18632/oncotarget.3723 (PMC4494954; doi:10.18632/oncotarget.3723)
Supplement: Supplementary file 1 [file oncotarget-06-12505-s001.pdf]

Inhibition of mutant IDH1 decreases D-2-HG levels without affecting tumorigenic properties of chondrosarcoma cell lines

Supplementary Material

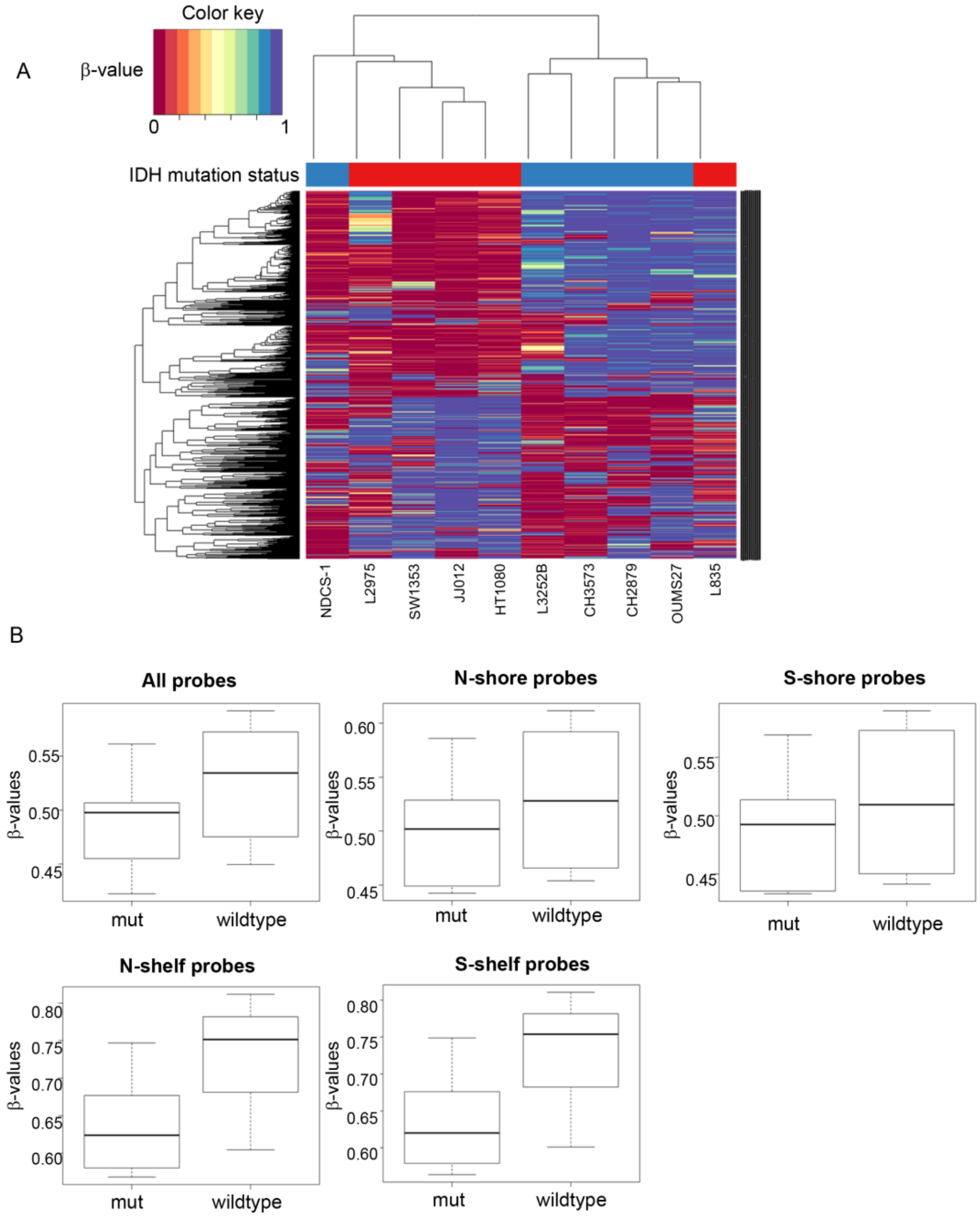

Supplementary figure 1: Comparison of methylome between IDH1/2 wildtype and mutant IDH1/2 chondrosarcoma cell lines.
A) Unsupervised hierarchical clustering for the top 2000 most differentially methylated CpG sites resulted in a wildtype (blue samples) and a mutation cluster (red samples). Interestingly, inclusion of all available probes resulted in more methylation in the IDH1/2 wildtype cell lines compared to the mutant IDH1/2 cell lines. B) Interestingly, methylation levels of all probes, probes in the shores and shelves were higher in the wildtype IDH1/2 cell lines compared to the mutant counterparts.

CH2879

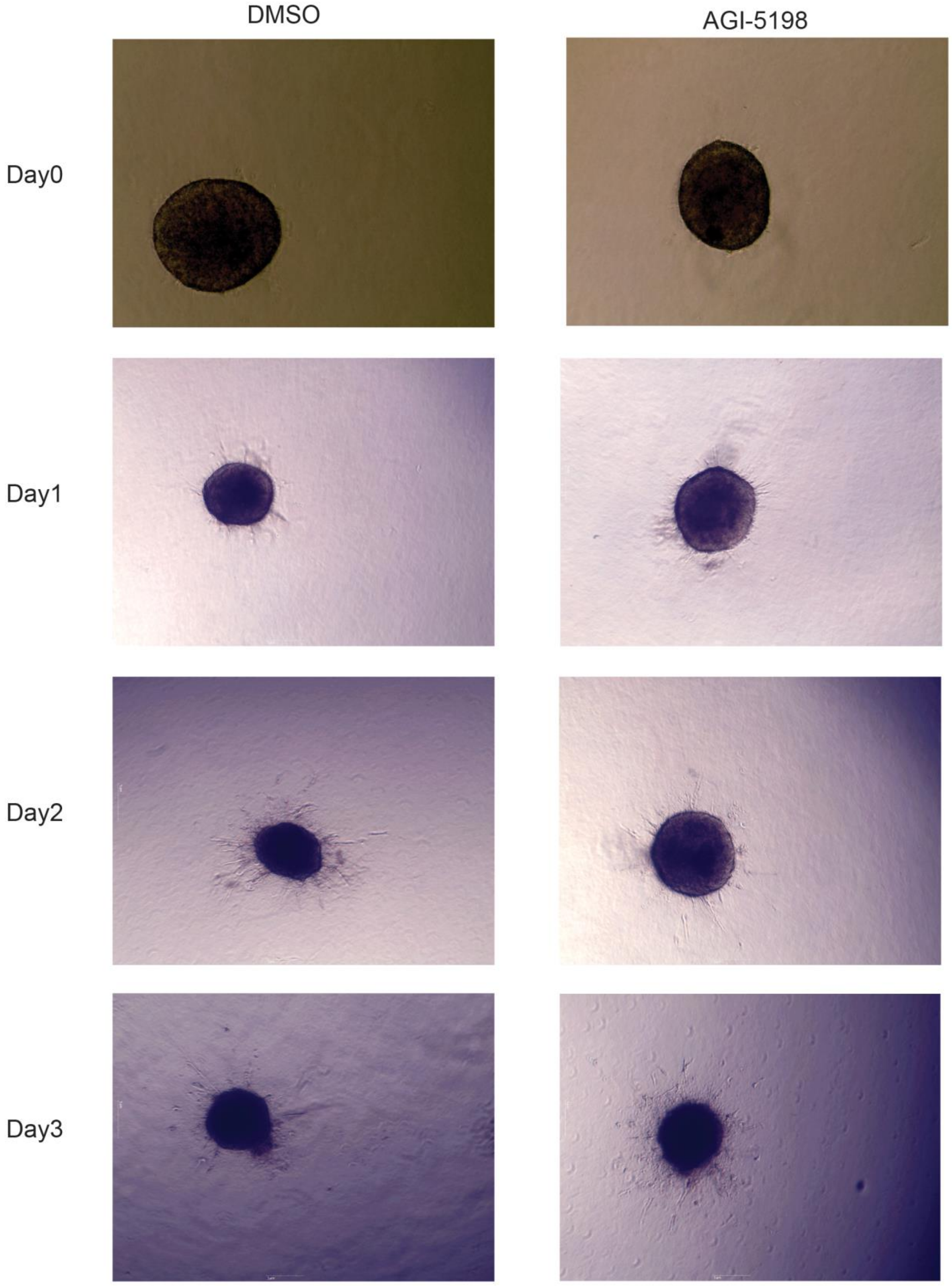

Supplementary figure 2: Effects of AGI-5198 treatment on migration in a 3D assay.
No differences in the ability to migrate in a 3D assay were observed between the IDH1/2 wildtype cell line CH2879, the mutant IDH1 cell lines JJ012 and HT1080 and the mutant IDH2 cell line SW1353. Furthermore, treatment with 10 μM AGI-5198 did not affect the ability of all cell lines to migrate. Pictures were taken one hour, one day, two days and three days after injection of cells into the matrix.

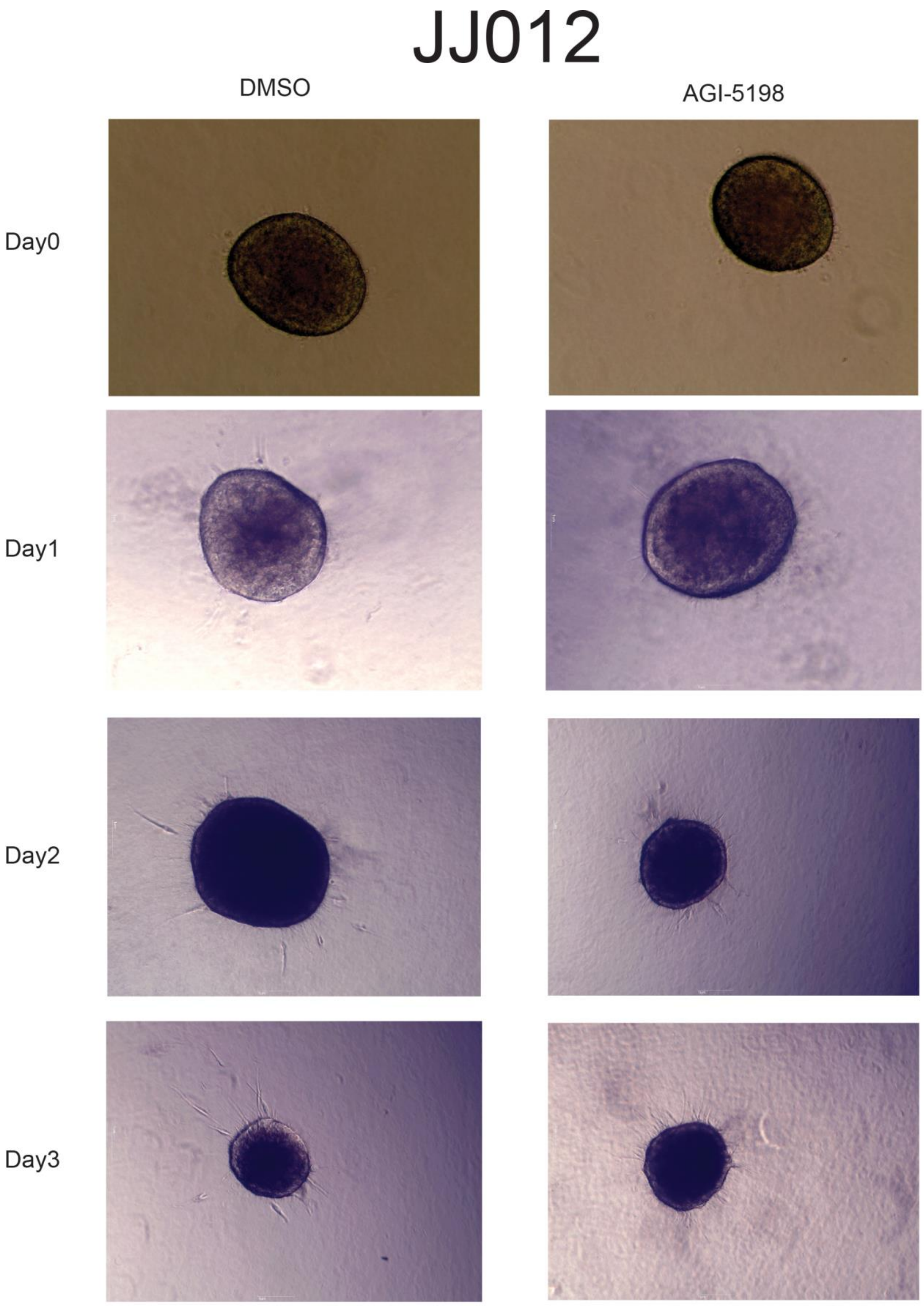

Supplementary figure 2: Effects of AGI-5198 treatment on migration in a 3D assay.
No differences in the ability to migrate in a 3D assay were observed between the IDH1/2 wildtype cell line CH2879, the mutant IDH1 cell lines JJ012 and HT1080 and the mutant IDH2 cell line SW1353. Furthermore, treatment with 10 μM AGI-5198 did not affect the ability of all cell lines to migrate. Pictures were taken one hour, one day, two days and three days after injection of cells into the matrix.

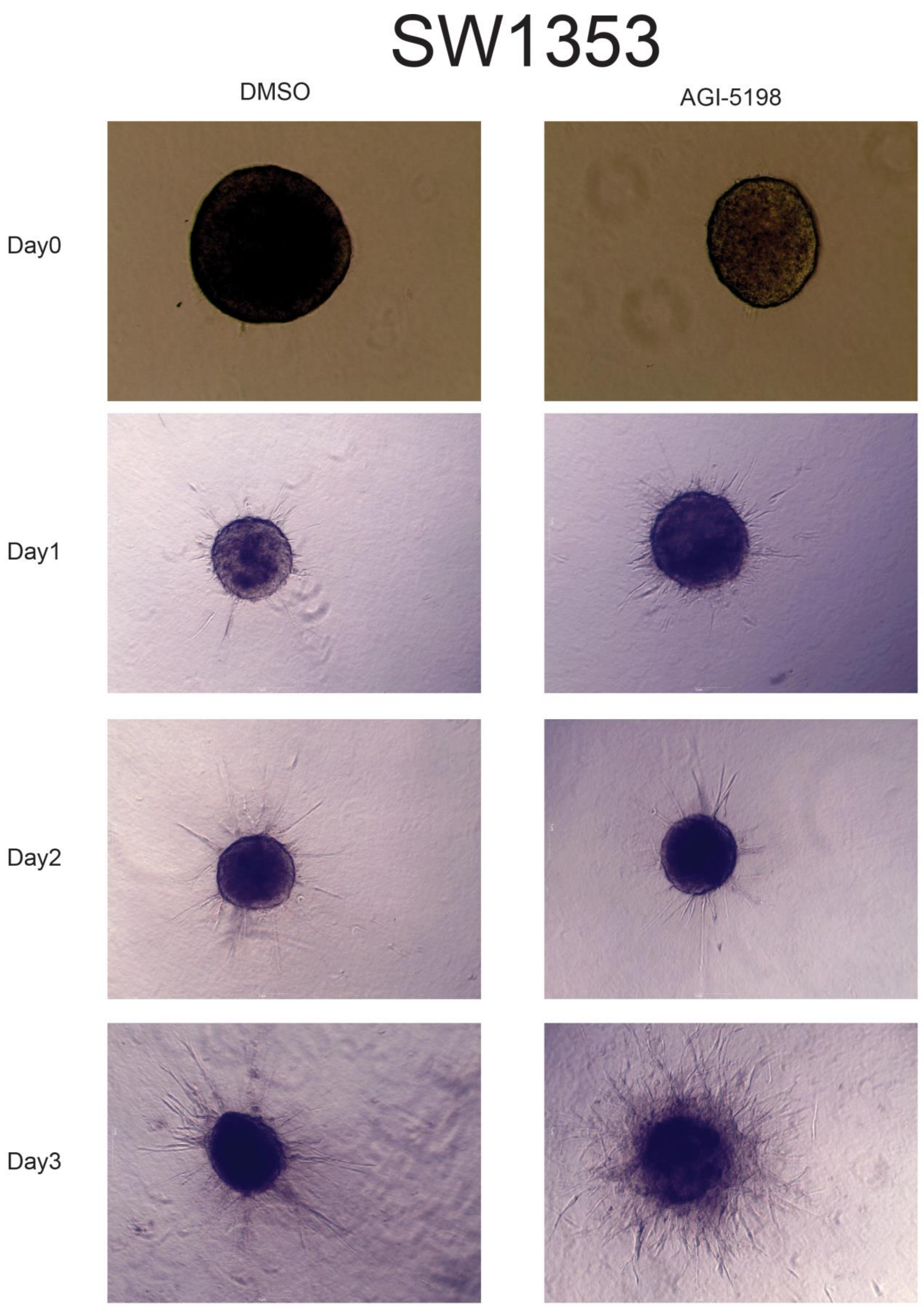

Supplementary figure 2: Effects of AGI-5198 treatment on migration in a 3D assay.
No differences in the ability to migrate in a 3D assay were observed between the IDH1/2 wildtype cell line CH2879, the mutant IDH1 cell lines JJ012 and HT1080 and the mutant IDH2 cell line SW1353. Furthermore, treatment with 10 µM AGI-5198 did not affect the ability of all cell lines to migrate. Pictures were taken one hour, one day, two days and three days after injection of cells into the matrix.

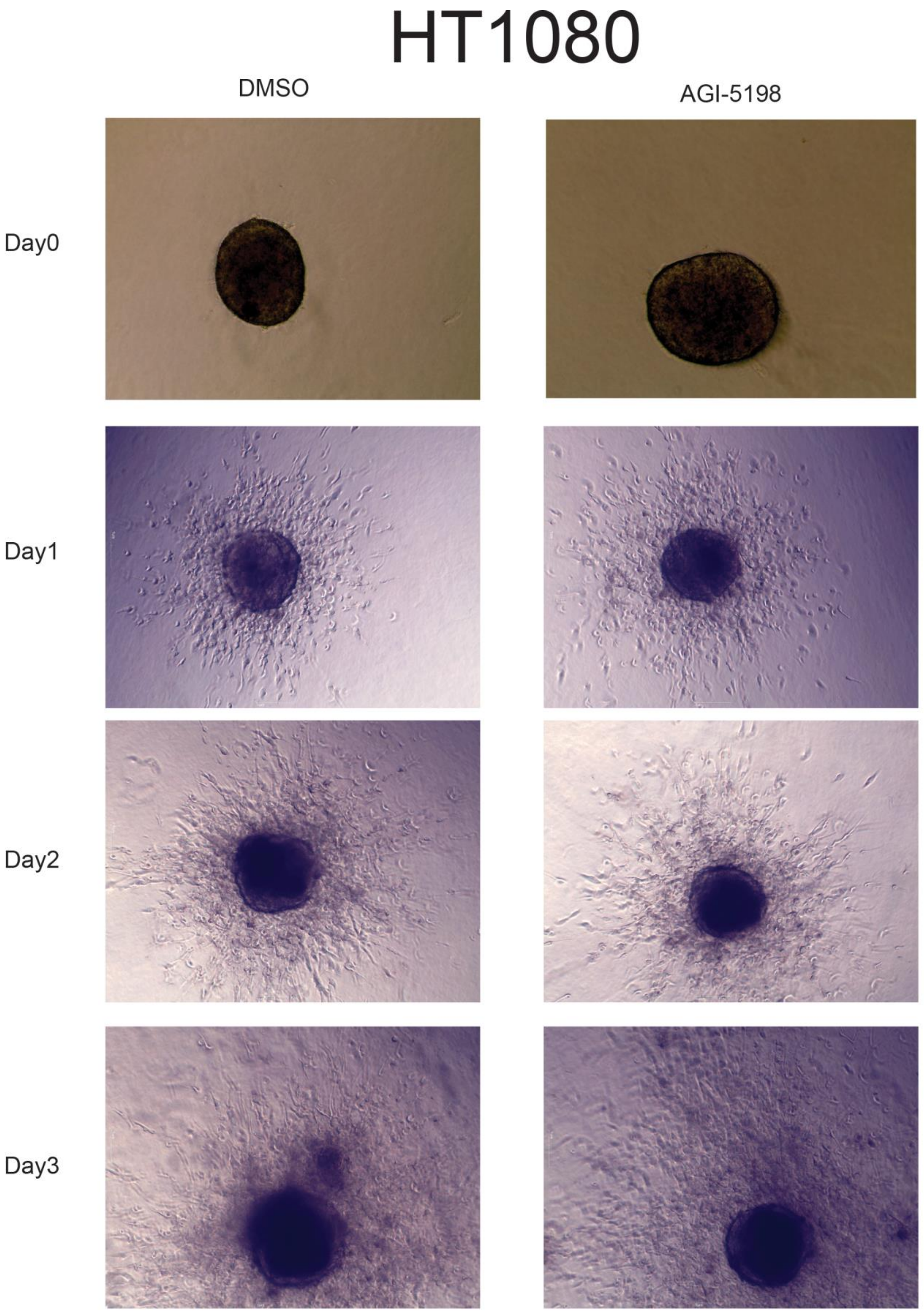

Supplementary figure 2: Effects of AGI-5198 treatment on migration in a 3D assay.
No differences in the ability to migrate in a 3D assay were observed between the IDH1/2 wildtype cell line CH2879, the mutant IDH1 cell lines JJ012 and HT1080 and the mutant IDH2 cell line SW1353. Furthermore, treatment with 10 μM AGI-5198 did not affect the ability of all cell lines to migrate. Pictures were taken one hour, one day, two days and three days after injection of cells into the matrix.

Supplementary table 1: Primers used for Quantitative Real Time PCR

**Gene symbol**	**Gene name**	**Forward primer**	**Reverse primer**
PPIA	CyclophilinA	TCATCTGCACTGCCAAGACTG	CATGCCTTCTTTCACTTTGCC
CPSF6	Cleavage and polyadenulation specific factor 6	AAGATTGCCTTCATGGAATTGAG	TCGTGATCTACTATGGTCCCTCTCT
GPR108	G-protein coupled receptor 108	AGATGCCCCTTTTCAAGCTCTAC	GCCATGAGCCAGTGGATCTTG
IHH	Indian hedgehog homolog	CCAATTACAATCCAGACATCATCTTC	GATAGCCAGCGAGTTCAGGC
PTCH1	Patched homolog	CCACGACAAAGCCGACTACAT	GCTGCAGATGGTCCTTACTTTTTC
SMO	Smoothened homolog	AGCGCAGCTTCCGGG	CAGTTCCAAACATGGCAAACAG
GLI1	Glioma-associated oncogene homolog	TGCAGTAAAGCCTTCAGCAATG	TTTTCGCAGCGAGCTAGGAT
GLI2	GLI-Kruppel family member GLI2	TTCTCCAACGCCTCGGAC	GTGGACCGTTTTCACATGCTT
GLUT1	Glucose transporter type 1	ATGAGAAGATGCCGATTTGG	TTTTCTGAGTGCCTGCTGTG
BNIP3	BCL2/Adenovirus E1B 19 kDa interacting protein	ACCCTCAGCATGAGGAACAC	AGCAGCAGAGATGGAAGGAA
EGLN3	Egl-9 family hypoxia inducible factor	AGCTACATGGTGGGATCCTG	ACTTCGTGTGGGTTCCTACG
ENO1	Enolase 1	GATCCCTTTGACCAGGATGA	CTGTGAGATCATCCCCCACT
VEGF	Vascular endothelial growth factor	TCTTCAAGCCATCCTGTGTG	ATCTGCATGGTGATGTTGGA
COL1a1	Collagen type 1a1	AAGACGAAGACATCCCACCAAT	GTCACAGATCACGTCATCGCA
COL2a1	Collagen type 2a1	GTCCTGCTGGTGGTCCTG	CGAGGACCTTGAGCACCTT
COL1a2	Collagen type 1a2	GCTGGAAAAGATGGTCGCAC	TAACCACCACCGCTTACACC
RUNX2	Runt-Related Transcription factor2	CAGAACCCACGGCCCTCCCT	CCCAGTGCCCCGTGTGGAAG
BGLAP	Osteocalcin	TGAGAGCCCTCACACTCCTC	ACCTTTGCTGGACTCTGCAC
OPN	Osteopontin	TTTCGCAGACCTGACATCC	GGCTGTCCCAATCAGAAGG
SPARC	osteonectin	GTGCAGAGGAAACCGAAGAG	TGTTTGCAGTGGTGGTTCTG
DIO2	Deiodinase iodothyronine type 2	TTCCAGTGTGGTGCATGTCTCTC	AGTCAAGAAGGTGGCATGTGG
